# Observing multifarious topological phase transitions with real-space indicator

**DOI:** 10.1515/nanoph-2021-0559

**Published:** 2021-11-26

**Authors:** Yong-Heng Lu, Yao Wang, Feng Mei, Yi-Jun Chang, Hang Zheng, Suotang Jia, Xian-Min Jin

**Affiliations:** Center for Integrated Quantum Information Technologies (IQIT), School of Physics and Astronomy and State Key Laboratory of Advanced Optical Communication Systems and Networks, Shanghai Jiao Tong University, Shanghai 200240, China; CAS Center for Excellence and Synergetic Innovation Center in Quantum Information and Quantum Physics, University of Science and Technology of China, Hefei, Anhui 230026, China; State Key Laboratory of Quantum Optics and Quantum Optics Devices, Institute of Laser Spectroscopy, Shanxi University, Taiyuan, Shanxi 030006, China; Collaborative Innovation Center of Extreme Optics, Shanxi University, Taiyuan, Shanxi 030006, China; Laboratory of Artificial Structures and Quantum Control (Ministry of Education), School of Physics and Astronomy, Shanghai Jiao Tong University, Shanghai 200240, China

**Keywords:** bulk-state-measurement, femtosecond laser direct written lattice, topological phase transition, topological photonics

## Abstract

First- and second-order topological phases, capable of inherent protection against disorder of materials, have been recently experimentally demonstrated in various artificial materials through observing the topologically protected edge states. Topological phase transition represents a new class of quantum critical phenomena, which is accompanied by the changes related to the bulk topology of energy band structures instead of symmetry. However, it is still a challenge to directly observe the topological phase transitions defined in terms of bulk states. Here, we theoretically and experimentally demonstrate the direct observation of multifarious topological phase transitions with real-space indicator in a single photonic chip, which is formed by integration of 324 × 33 waveguides supporting both first- and second-order topological phases. The trivial-to-first-order, trivial-to-second-order and first-to-second-order topological phase transitions signified by the band gap closure can all be directly detected via photon evolution in the bulk. We further observe the creation and destruction of gapped topological edge states associated with these topological phase transitions. The bulk-state-based route to investigate the high-dimensional and high-order topological features, together with the platform of freely engineering topological materials by three-dimensional laser direct writing in a single photonic chip, opens up a new avenue to explore the mechanisms and applications of artificial devices.

## 1 Introduction

Topological phases, the core of fundamental description of characterizing the states of matter with global wave function [1, 2], have been rapidly developed for searching novel topological materials and applied in a variety of artificial topological systems [3–8]. Originating from the integer quantum Hall effect [9, 10], topological insulators, harnessing different topological phases [11] with bulk and edge properties, inherently enable the superior capabilities of lossless flowing of charges and information along surface. In addition to the fundamental physics, the unique robustness of topological phases and their transitions are predicted to be the promising candidate for fault-tolerant quantum computing [12] and superconductors [2]. Different from the conventional transition of phases of matter accompanied by the broken symmetry [13], the transition among different topological phases is particularly characterized by the discontinuous changes of topological invariants [3] with the closed band gap [1, 2], which still remains a long-standing challenge to detect.

Drawing inspiration from the topology in condensed matter, analogous effects in electrons have been elegantly mapped to photons [3–5], leading to advances related to the fascinating phenomena of robust unidirectional propagation of light against disorder and defects [14–20]. It holds enormous promise for the next generation of integrated circuits for routing light in classical [21] and quantum region [22–24]. Particularly, the exploration of first- and second-order topological phases [25–27] provides the topologically protected one-way channels in edges and corners for photonics systems, including topological insulator edge states [14–20] and second-order topological insulators [28–32] in multidimensional systems. It can be utilized for quantum information processing [22, 33], [34], [35] and developing the inherently robust photonic devices [36–39].

Compared with the wide investigation of topological phases with topologically protected edge and corner states [14], [15], [16], [17], [18], [19], [20, 28], [29], [30], [31], [32], the topological phase transitions defined in terms of bulk states in the photonic systems are rarely examined. For photons, it is challenging to resolve the wave functions defined in momentum space with bulk features of the systems. Recently, several progresses in exploring topological phase transition in photonic system have been proposed and implemented in 1D system, based on observing subgap states with adiabatically smooth edge [40, 41], dynamics with discrete quantum walk [42–46] and loss localized on sub-lattices [47, 48]. However, while entering into the realm of high dimensional and high order topology, the manipulation and manifestation of bulk topology for topological phase transition have not been explored yet.

Here, we theoretically and experimentally observe multifarious topological phase transitions with coexistence of three phases, including trivial phases, first- and second-order topological insulator phases. We design and fabricate 33 topologically-differential lattices, each containing 18 × 18 sites, which are all integrated in one chip using the femtosecond laser direct writing technique with flexible site-engineering fashion. The transition with the band gap closure in energy-momentum space is directly detected by the indicator of bulk propagation in real space.

## 2 Results

### 2.1 Model of TPTI

**Figure 1:**
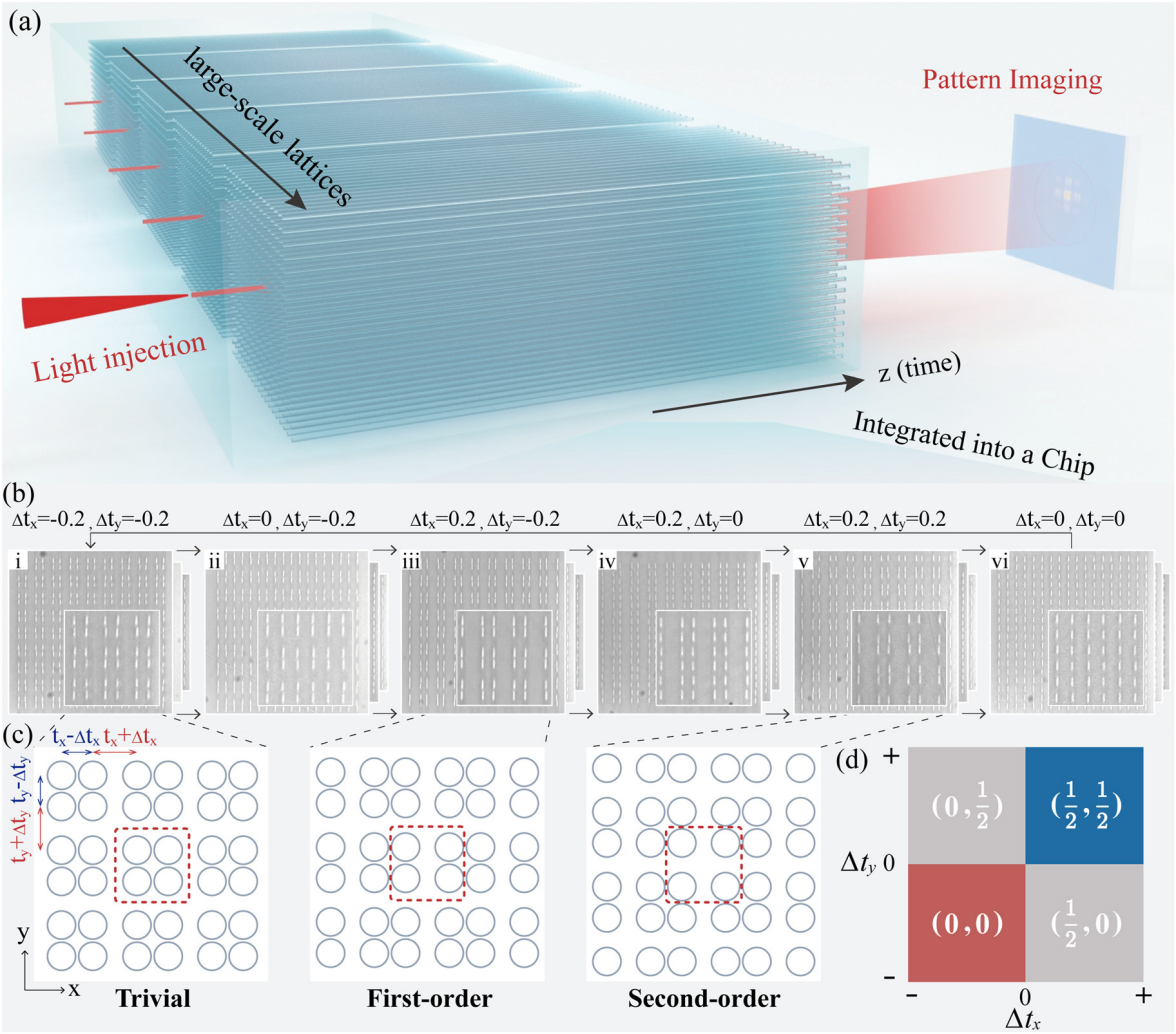
Schematic of observing topological phase transition in highly integrated lattices. (a) The photons are injected into the entrance waveguide in the central unit cell. All the large-scale topologically-differential lattices, possessing different topological phases, are integrated in a chip with the well-locked environment. (b) The micro crosssection of the topologically-differential lattice with gradually varying distortion. (c) Trivial phase, FOTI phase and SOTI phase are supported in the fabrication samples. The parameter *t*_*x*,*y*_ = 0.3 and Δ*t*_*x*,*y*_ is smoothly modulated from −0.2 to 0.2. The unit of the coupling constants is [mm^−1^]. (d) Topological phase diagram characterized by the wave polarization. Trivial phase, FOTI phase and SOTI phase are marked in red, gray and blue regions, respectively.

The topological lattices are all integrated in one femtosecond laser-written borosilicate silica chip [49, 50], as shown in Figure 1a. The two-dimensional (2D) topological model promoted from the one-dimensional dimer chain [51] could be described by

(1) $$\begin{array}{ll} H & =\underset{m,n}{\sum }\left(t_{x}+{\left(-1\right)}^{m}\Delta t_{x}\right)C_{m+1,n}^{†}C_{m,n}\\ & \;+\left(t_{y}+{\left(-1\right)}^{n}\Delta t_{y}\right)C_{m,n+1}^{†}C_{m,n}+\text{H}.\text{c}. \end{array}$$

where *C* ^†^(*C*) is the creation (annihilation) operator of site (*m*, *n*) along (*x*, *y*) directions and *t*_*x*,*y*_ represents the average hopping strength. The combination of Peierls distortions (−1)^*m*,*n*^ and the dimerization Δ*t*_*x*,*y*_ determines the strong and weak couplings for intra-cell and inter-cell coupling for two directions, which can be modulated by the spacing between the nearest waveguides in the photonic lattices (see Figure 1b and c). The corresponding Hamiltonian in the momentum space can be expressed as a 4 × 4 matrix H(k) , where **k** = (*k*_*x*_, *k*_*y*_). The elements $H_{12}=H_{34}=t_{x1}+t_{x2}\;{\text{e}}^{\text{i}k_{x}}$ , $H_{13}=H_{24}=t_{y1}+t_{y2}{\text{e}}^{-\text{i}k_{y}}$ ; *H*_21_, *H*_42_, *H*_31_, *H*_43_ are the Hermitian conjugate terms; the others are zero terms. Here, *t*_*x*1(*y*1)_ and *t*_*x*2(*y*2)_ represent *t*_*x*(*y*)_ − Δ*t*_*x*(*y*)_ and *t*_*x*(*y*)_ +Δ*t*_*x*(*y*)_, respectively. Owing to the coexistence of time-reversal and inversion symmetries, our model (see Figure 1b and c) exhibits a novel topological phase with the vanished Berry curvature [52, 53].

Using the norm of the polarization in crystalline dielectrics [54], the phases can be characterized by the wave polarization

(2) $$Q=\frac{1}{2\pi }\int \text{d}k_{x}\;\text{d}k_{y}\mathrm{Tr}\left[A\left(k_{x},k_{y}\right)\right]$$

where $A=\left\langle \psi \left|i\partial_{k}\right|\psi \right\rangle$ is the integration of the Berry connection over the first BZ [52, 53]. The wave polarization here is totally determined by the bulk property of the systems [52]. The numerical results for the wave polarization shown in Figure 1d indicate that there are three different topological phases in our system. Specifically, when Δ*t*_*x*_ < 0 and Δ*t*_*y*_ < 0, *Q* = (0, 0), the system is in the trivial topological insulator phase; when Δ*t*_*x*_ > 0 and Δ*t*_*y*_ > 0, $Q=\left(\frac{1}{2},\frac{1}{2}\right)$ , the system is in the second-order topological insulator (SOTI) phase [28, 29]; while for Δ*t*_*x*_ or Δ*t*_*y*_ < 0, the system is in the first-order topological insulator (FOTI) phase. Thus, our system supports three topological phase transitions which can be engineered and driven by modulating the distortion Δ*t*_*x*(*y*)_.

**Figure 2:**
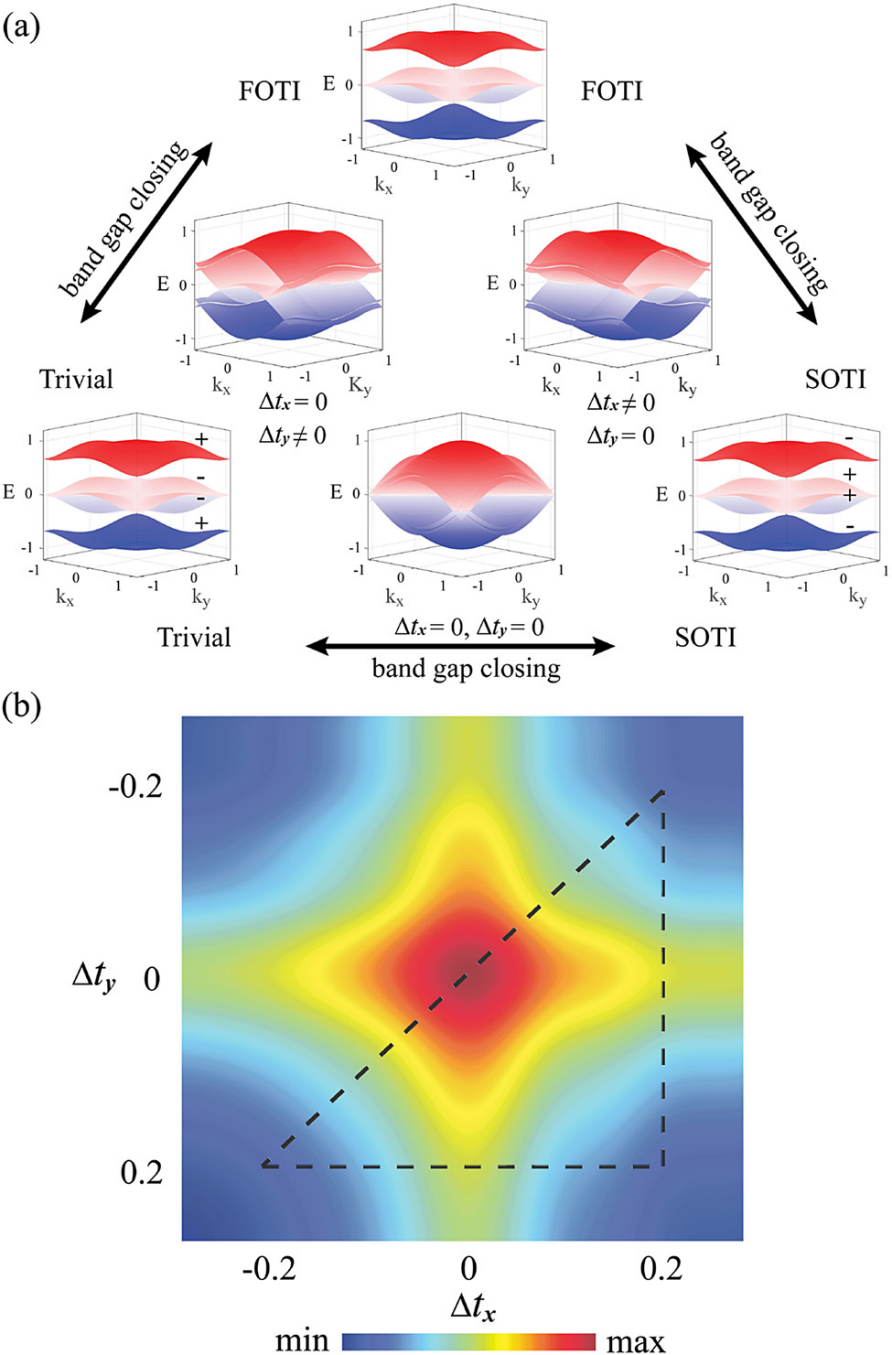
Topological phase transition and its indicator. (a) The band structures in topological phase transition. The arrows indicate the phase transition among trivial, FOTI and SOTI phases. (b) The simulated results of TPTI as a function of Δ*t*_*x*,*y*_. The dash line shows the phase transition trajectories detected in experiment.

Topological phase transition is always accompanied with the energy band gap closing process [1, 2]. We numerically draw the energy band structure varying with Δ*t*_*x*_ and Δ*t*_*y*_ under the periodic boundary condition (depicted in Figure 2a). There are four energy bands in our model. For the topological phase transition between the trivial phase, *Q* = (0, 0), and the FOTI phase, $Q=\left(\frac{1}{2},0\right)$ , the energy band closure occurs when Δ*t*_*x*_ = 0, where the second (third) energy band touches the first (fourth) energy band along *k*_*x*_ direction. There is also phase transition in the process between the FOTI phase, $Q=\left(\frac{1}{2},0\right)$ , and the SOTI phase, $Q=\left(\frac{1}{2},\frac{1}{2}\right)$ with the band gap closure emerging at Δ*t*_*y*_ = 0. For the phase transition process between the trivial phase, *Q* = (0, 0), and the SOTI phase, $Q=\left(\frac{1}{2},\frac{1}{2}\right)$ , the four energy bands degenerately coincide at four corners of the first Brillouin zone when Δ*t*_*x*,*y*_ = 0. Moreover, the phase transition also associates with band inversion process [52, 53].

To unravel and visualize the aforementioned bulk band gap closure from momentum space to real-space observable, we employ the expected value of the square of position operator in long-time limit as an indicator. The square of position operator in our model is defined as

(3) $$\begin{array}{ll} r^{2} & =\overset{N}{\underset{x,y=1}{\sum }}\left(x^{2}+y^{2}\right)\left(P_{2x-1,2y-1}+P_{2x-1,2y}\right.\\ & \;\left.+P_{2x,2y-1}+P_{2x,2y}\right) \end{array}$$

where $P_{m,n}=c_{m,n}^{†}c_{m,n}$ is the photon population probability of the site (*m*, *n*) and index (*x*, *y*) is the position of unit cell. The injection of photons into one of the waveguides in the middle unit cell of lattices (far from boundary) can be regarded as the excitation of bulk states.

After propagating a long distance *z*, the diffraction behavior of photons can be characterized by the expected value of the square of position operator. This process is measured by: $\bar{r^{2}}\left(z\right)=\left\langle \psi \left(z\right)|r^{2}|\psi \left(z\right)\right\rangle$ , which reflects the generalized photon density centre located at the 2D lattices. Based on the density centre, the topological phase transition indicator (TPTI) can be defined as: $S_{t}=\bar{r^{2}}/z^{2}$ for 2D systems. It characterizes the diffraction range of photons in waveguide lattice. Furthermore, the TPTI can be analytically derived as

(4) $$S_{t}=\left\{\begin{array}{cc} \frac{{\left(t_{x}-\Delta t_{x}\right)}^{2}+{\left(t_{y}-\Delta t_{y}\right)}^{2}}{2},\; & \Delta t_{x}>0,\;\Delta t_{y}>0\\ \frac{{\left(t_{x}+\Delta t_{x}\right)}^{2}+{\left(t_{y}+\Delta t_{y}\right)}^{2}}{2},\;\; & \Delta t_{x}<0,\;\Delta t_{y}<0 \end{array}\right.$$

where TPTI is the coupling-strength dependent quantity (see the Supplementary Material Section II for the relationship between topological phase transitions and TPTI in detail).

Simulated TPTI for characterizing the topological phase transition as a function of Δ*t*_*x*,*y*_ are shown in Figure 2b. The value of TPTI is higher when Δ*t*_*x*,*y*_ approaches to zero. For topological phase transition between trivial phase and FOTI phase along horizontal routes (FOTI phase and SOTI phase along vertical routes), the TPTI possesses a peak pinned at the turning point of Δ*t*_*x*_ = 0 (Δ*t*_*y*_ = 0). When trivial phase evolves to SOTI phase along the diagonal routes with simultaneous modulation for two directions, there is a higher peak located at the transition point of Δ*t*_*x*,*y*_ = 0 (see the Supplementary Material Section III for properties of TPTI in detail). In this way, the topological phase transition can be directly mapped to the peak indicator of TPTI observed in real space.

### 2.2 Experimental observation of TPTI

In our experiment, to observe the topological phase transitions between multifarious topological phases, we implement integrated topological photonic lattices in an on-demand fashion by femtosecond laser direct writing (more details are shown in the Supplementary Materials). We set the case of trivial-to-first-order (first-to-second-order) by varying Δ*t*_*x*_ from −0.2 to 0.2 with a step of $0.04\left(\right.\Delta t_{y}$ from −0.2 to 0.2 with a step of 0.04) and remaining Δ*t*_*y*_ = −0.2 (Δ*t*_*x*_ = 0.2). We then detect the transition process between SOTI and trivial phases by simultaneously modulating Δ*t*_*x*_ and Δ*t*_*y*_, both from 0.2 to −0.2 with a step of −0.04. Such that, each transition process is monitored by 11 samples containing 18 × 18 sites (9 × 9 unit cells) individually. It means that all the 33 lattices and up to ten thousands waveguides are integrated in one photonic chip, which guarantees each topologically-differential lattice with different topological phases placed in a well-locked environment. After photons launched into the middle unit cell of the lattice, the intensity distribution can be directly captured by an imaged camera system (shown in Figure 1a).

**Figure 3:**
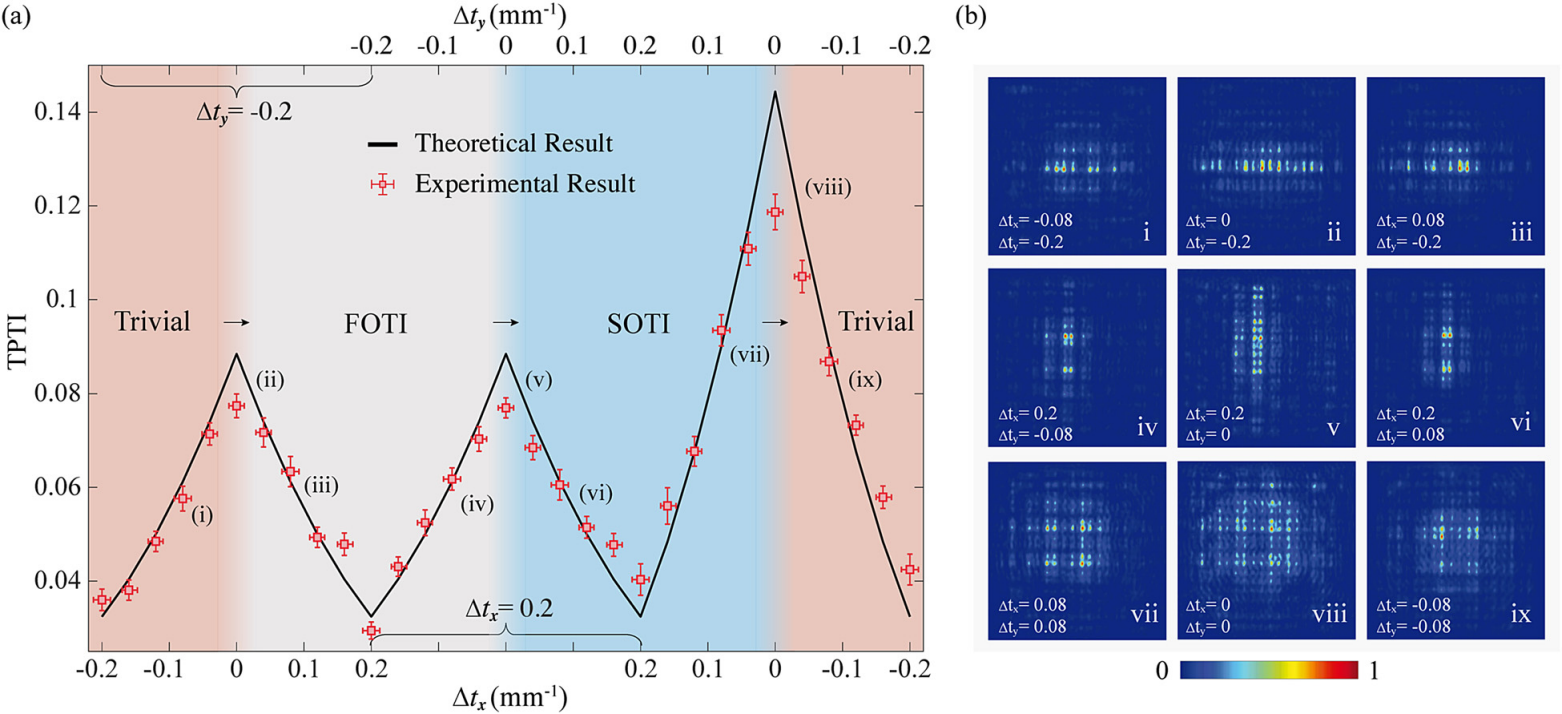
Experimental measurement of topological phase transition indicator. (a) The measured TPTI as a function of Δ*t*_*x*_ and Δ*t*_*y*_ along the triangular trajectory shown in Figure 2(b). There are three peaks at the phase transition points where the system undergoes topological phase transitions. The evolution length for all the waveguide lattices is 14 mm. The error bar is originated from the fabrication shift of the waveguide lattices. (b) Output photon distribution of the waveguide lattices with the coupling strengths chosen as (i–ix) marked in (a), corresponding to (i) trivial phase, (ii) gap closing separating trivial and FOTI phases, (iii) FOTI phase, (iv) FOTI phase, (v) gap closing separating SOTI and FOTI phases, (vi) SOTI phase, (vii) SOTI phase, (viii) gap closing separating SOTI and trivial phases and (ix) trivial phase. The coupling strength *t*_*x*,*y*_ = 0.3 mm^−1^. The unit of the coupling constants is mm^−1^.

The experimental results of TPTI for three cases are shown in Figure 3. The intensity distribution cannot show any clues for distinguishing the occurrence of topological phase transition with clear-cut evaluation. But there are three peaks for TPTI when the system suffers three topological phase transitions among trivial, FOTI and SOTI phases (see Figure 3a). During modulation of Δ*t*_*x*,*y*_ on the photonic chip, peaks of TPTI sign the topological phase transition points with band gap closure. It turns out that we can monitor the sudden change of the topology by TPTI in the topological phase transition process. In addition, the peak in the third transition case for SOTI to trivial phase is distinctly higher, as expected. Therefore, the band gap closing points defined in the momentum space for characterizing the topological phase transition is experimentally observed through the real space observable TPTI.

One essential feature of topological phase transition is the creation and destruction of topologically protected edge states. To visualize the wave function for the edge states, we launch photons into the boundaries of the lattices and see the intensity distribution. The localization of the edge states can be quantified by generalized return probability [40] $\xi =\left(\sum_{i=n}^{n+\Delta }|\psi_{i}{|}^{2}\right)/\left(\sum_{i=1}^{N}|\psi_{i}{|}^{2}\right)$ , where *N* is the total site number of lattice, *n* is the site along the exciting boundary of the lattice and Δ is the index interval between the neighboring sites along the excited boundary.

**Figure 4:**
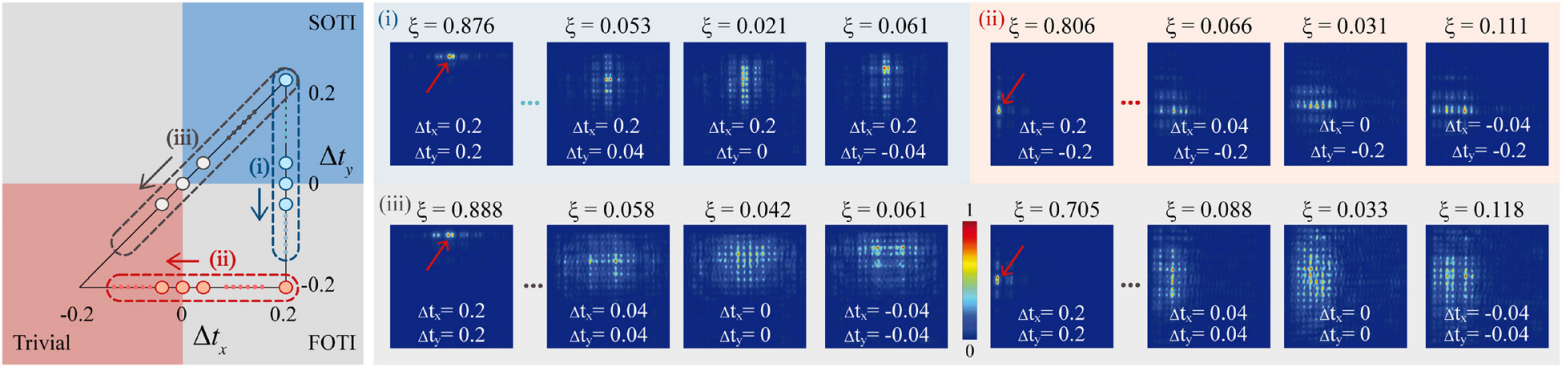
Experimental observation of creation and destruction of topological edge states. There are three detected trajectories for observing the creation and destruction of topological edge states under the modulation of Δ*t*_*x*,*y*_. The process from SOTI to FOTI phases, from FOTI to trivial phases and from SOTI to trivial phases is marked by blue, red and gray dash lines, respectively. The red arrows indicate the launching positions

We drive SOTI phase into FOTI phase by only decreasing Δ*t*_*y*_ from 0.2 to −0.04 and remain Δ*t*_*x*_ = 0.2, corresponding to the transition from $Q=\left(\frac{1}{2},\frac{1}{2}\right)$ to $Q=\left(\frac{1}{2},0\right)$ based on Eq. (2) (see Figure 1d). The changes of wave polarization along *y* direction imply the creation and destruction for the corresponding edge states (more details are shown in the Supplementary Materials Section IV). As shown in Figure 4i, the top localized edge state emerges with high return probability and then disappears with low return probability. For the case of transition from FOTI phase $\left(\right.Q=\left(\frac{1}{2},0\right)$ to trivial phase (*Q* = (0, 0)) by only decreasing Δ*t*_*x*_ from 0.2 to −0.04 and remain Δ*t*_*y*_ = −0.2, there is a sudden change for wave polarization along the *x* direction. The creation and destruction of left localized edge states are observed during the transition (see Figure 4ii). We also simultaneously drive both Δ*t*_*x*_ and Δ*t*_*y*_ from 0.2 to −0.04, which corresponds to the transition from $Q=\left(\frac{1}{2},\frac{1}{2}\right)$ to *Q* = (0, 0). There are sudden changes for both wave polarizations along the two directions. As shown in Figure 4iii, both top and left edge states simultaneously localize in the boundaries and then diffract into the bulk, accompanied with the transition from SOTI phase to trivial phase. In addition, it can be noted that, in the region close to the phase transition points (Δ*t*_*x*_ = 0 or Δ*t*_*y*_ = 0), the exciting edge states all tend to diffuse with low return probability, which shows insensitivity to the turning points. However, the demonstrated TPTI shown in Figure 3 is much sensitive to all the turning points, which precisely distinguishes the occurrence of the transition among SOTI, FOTI and trivial phases.

## 3 Conclusions and discussions

In summary, the observation of topological phase transition with full control of bulk topology and freely engineering fashion, including topological lattices with trivial, FOTI and SOTI phases, is fundamentally essential for understanding high-dimensional and high-order topological physics. The developed TPTI approach can directly identify the multifarious phase transitions based on the photon evolution pattern in 2D systems, instead of the complex statistical detection with multi-step and multi-time [42, 44, 45, 55]. The signal of topological phase transition is based on the excitation of bulk states with engineering of the spatial geometry, free of constructing adiabatically smooth boundary with stringent demand between topologically distinct phases [40].

The successful observation of the photon propagation mapping the band gap closure in the Hermitian system here may inspire the future exploration in the non-Hermitian systems [56], such as, metal-insulating phase transition in the non-Hermitian quasicrystals [57] and phase transition in parity-time-symmetric crystal [58]. Finally, our findings pave a novel avenue for exploring topological phase transitions in other artificial systems with high-dimension and high-order beyond photonics, such as phononic [59–62], microwave [63], electrical circuits [64, 65] and plasmon-polaritonic [66].

## Supplementary Material

Supplementary Material Details

Supplementary Material Details
