# Supplementary material for: Observing multifarious topological phase transitions with real-space indicator
Source: Nanophotonics. 2021 Nov 26;11(1):153–60. doi: 10.1515/nanoph-2021-0559 (PMC11501473; doi:10.1515/nanoph-2021-0559)
Supplement: Supplementary file 1 — Supplementary Material Details [file j_nanoph-2021-0559_suppl_001.pdf]

Supplementary Materials: Observing Multifarious Topological Phase Transitions in a Single Topological Chip

I. Fabrication and modulation

In the main text, we study the topological phase transition in the integrated photonic system holding the high level of control over their parameters. The arrays of photonic lattices, composed of the single-mode waveguides, are all well fabricated and integrated in a borosilicate glass chip (refractive index $n_0 = 1.514$) using the femtosecond laser direct writing technology (see Fig.S3). The propagating light in one waveguide tunnels to the neighbouring ones via the overlap between evanescent modes of the adjacent waveguides, which leads to coupling and interaction. The coupling amplitude between adjacent waveguides can be controlled by modulating the spacing between them, as shown in Fig.S3a. Here, we use spatial light modulator (SLM) to shape the pumping laser beam with creating burst trains in both time and spatial domain. The SLM is fed by a 513 nm femtosecond laser (up conversion from 1026 nm pump laser; 10 W; 290 fs pulse duration; 1 MHz repetition rate).

Here, the phase transition is induced by modulating $t_{x,y}$ without tuning of the on-site potential. Therefore, The waveguides were all identical in refraction index via keeping the constant velocity of 10 mm/s and the constant pumping power. The evolution length of the lattices is $20mm$, which is equivalent to almost 66 coupling lengths. Each lattice contains 324 sites with the size of 18×18 (see Fig.S3b). To avoid the potential impact caused by depth-dependent coupling changes in the large size lattice, the uniformity of waveguides at different depths needs to be improved. The uniformity and depth-independence of the large array can be guaranteed by the SLM and power compensation.

Moreover, to demonstrate the topological phase transition in the 2D systems, we investigate three phase transition processes between three topological phases. The average hopping strength of $t_{x,y}$ is $0.3mm^{-1}$ in our system. Phase transition process between trivial phase and first order topological insulator phase is realized by modulating the control parameters Δt_x from $-0.2mm^{-1}$ to $0.2mm^{-1}$ with step of $0.04mm^{-1}$ and keeping $\Delta t_y = -0.2mm^{-1}$. For phase transition between first order topological insulator phase and second order topological insulator phase, the distortion parameter Δt_y is varying from $-0.2mm^{-1}$ to $0.2mm^{-1}$ with step of $0.04mm^{-1}$ and keeping $\Delta t_x = 0.2mm^{-1}$. For phase transition between second order topological insulator phase and trivial phase, the distortion parameter Δt_x and Δt_y is both varying from $0.2mm^{-1}$ to $-0.2mm^{-1}$ with step of $-0.04mm^{-1}$, simultaneously. In addition, each lattice contains 324 sites, which means there are up to ten thousand waveguides fabricating and integrating in our chip. Although all the parameters and environment during femtosecond laser direct-writing process have been optimized and locked, the small disorder, induced by the fabrication shifts, inevitably exists in each waveguide and each lattice. The topological phase transition is induced by modulating $\Delta t_{x,y}$ and the coupling is determined by the spacing between adjacent waveguides. Thus, the shift of spacing between waveguides is the main error source in our system. The error bar of experimental results in Fig.3 in the main text is originated from the uncertainty of waveguide spacing, which is induced by the limitation of fabrication accuracy. It should be noted that the relation between coupling coefficients and spacing is non-linear. During the increasing of spacing, the coupling strength decreases dramatically at the beginning but decreases smoothly at the end. It means that in different spacing region, the same fabrication shift of the spacing can lead to different deviations of the coupling strength.

II. The relationship between topological phase transitions and TPTI

In this section, we will demonstrate that the topological phase transition in 2D systems discussed in the main text can be directly detected by the photon propagation in the two-dimensional photonic lattice. Here, the 2D system can be described by the Hamiltonian using the tight-binding approximation:

$$H = \sum_{m,n} (t_x + (-1)^m \Delta t_x)\, C^{\dagger}_{m+1,n} C_{m,n} + (t_y + (-1)^n \Delta t_y)\, C^{\dagger}_{m,n+1} C_{m,n} + H.c. \tag{S1}$$

where the position of each sites in the two-dimensional photonic lattice is labeled as (m, n) along (x, y) direction. $t_{x,y}$ represents the average hopping strength in the system and the combination of $(-1)^{m,n}$ and $\Delta t_{x,y}$ represents

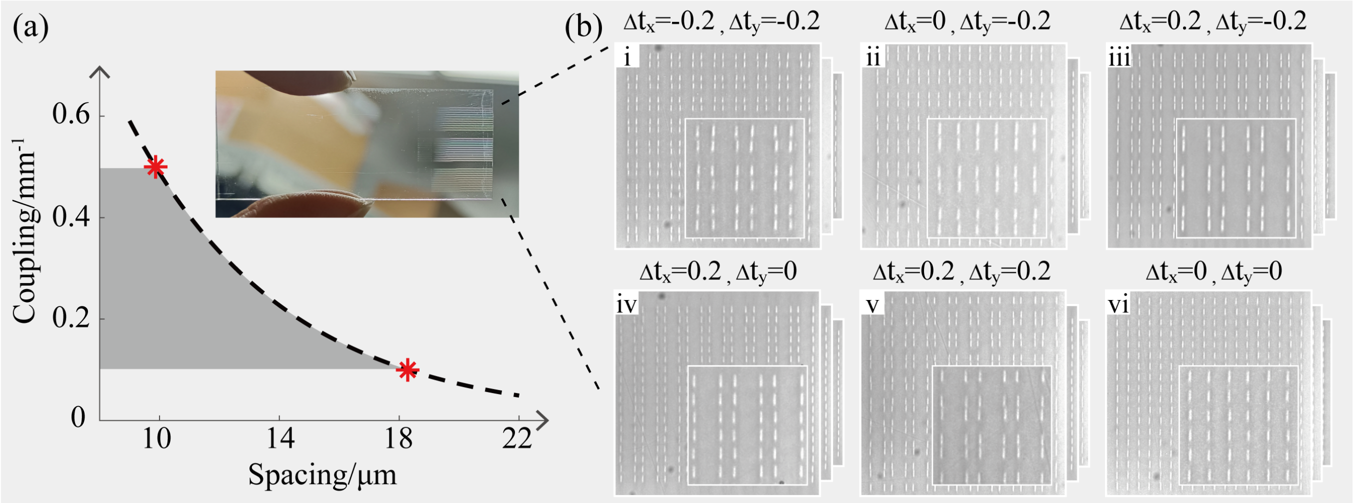

FIG. S1. **Fabrication of the integrated lattices** (**a**) Characterized coupling coefficients and their exponential dependence on the waveguide spacing. The gray region marks the accessible range of parameters in experiment. The bounds of the range of parameters are marked by two red stars. Inset shows the integrated chip containing 33 topologically-differential lattices. (**b**) The micro crosssection of the topologically-differential lattice with gradually varying distortion. The parameter $t_{x,y} = 0.3$, $\Delta t_{x,y}$ is smoothly modulated from -0.2 to 0.2. The unit of the coupling constants is $[1/mm]$.

the distortion modulation for couplings of intra-cell and inter-cell along two directions. By transformation in the momentum space, we can get

$$H(k) = \begin{pmatrix} 0 & J_1 + J_2 e^{ik_x} & J_1 + J_2 e^{-ik_y} & 0 \\ \left(J_1 + J_2 e^{ik_x}\right)^* & 0 & 0 & J_1 + J_2 e^{-ik_y} \\ \left(J_1 + J_2 e^{-ik_y}\right)^* & 0 & 0 & J_1 + J_2 e^{ik_x} \\ 0 & \left(J_1 + J_2 e^{-ik_y}\right)^* & \left(J_1 + J_2 e^{ik_x}\right)^* & 0 \end{pmatrix} \tag{S2}$$

where $t_{x1(y1)}$ and $t_{x2(y2)}$ represents $t_{x(y)} - \Delta t_{x(y)}$ and $t_{x(y)} + \Delta t_{x(y)}$, respectively.

In the following, to further examine how the topological phase transition with the band gap closing point defined in the momentum space discussed in the main text can be detected by the photon propagation in the 2D lattice, we employ the expect value of the square of the 2D position operator in the long-time limit as an indicator of topological phase transitions. The square of the 2D position operator in our model is defined as

$$r^2 = \sum_{x,y=1}^{N} (x^2 + y^2)(P_{2x-1,2y-1} + P_{2x-1,2y} + P_{2x,2y-1} + P_{2x,2y}) \tag{S3}$$

where (x, y) represents the position label of each unit cell along two directions in the two-dimensional photonic lattice. $P_{m,n} = c^{\dagger}_{m,n} c_{m,n}$ is the photon population probability in the waveguide lattice site (m, n). We inject photons into the middle waveguide to excite the bulk state of the system. Thus, the initial exciting state of the 2D photonic lattice can be represented by $|\psi(0)\rangle$. After evolution for certain distance z, the square of the 2D position operator associated in the waveguide lattice can be described as

$$\bar{r^2}(z) = \langle \psi(0)| e^{iHz} r^2 e^{-iHz} |\psi(0)\rangle. \tag{S4}$$

Furthermore, we define the topological phase transition indicator (TPTI) in 2D systems, which is expressed as

$$S_t = \bar{r^2}/z^2 \tag{S5}$$

By using Fourier transform, the TPTI in real space can be transform to the momentum space, we can further get

$$S_t = \frac{1}{4\pi^2} \int_{-\pi}^{\pi} dk_x \int_{-\pi}^{\pi} dk_y \langle \psi(0)| e^{i\hat{h}(k_x)z} ((i\partial_{k_x})^2 + (i\partial_{k_y})^2) e^{-i\hat{h}(k_x)z} |\psi(0)\rangle / z^2. \tag{S6}$$

In the long propagation distance limit, the terms proportional to $1/z$ can be omitted. Therefore, the above form can be simplified, which can be described by:

$$S_t = \frac{1}{2\pi}\int_{-\pi}^{\pi} dk_x(\partial_{k_x}E_x)^2 + \frac{1}{2\pi}\int_{-\pi}^{\pi} dk_x(\partial_{k_y}E_y)^2 \tag{S7}$$

where $E_x = \sqrt{h_{x1}^2 + h_{x2}^2}$ and $E_y = \sqrt{h_{y1}^2 + h_{y2}^2}$, with $h_{x1(y1)} = t_{x1(y1)} + t_{x2(y2)}\cos(k_{x(y)})$ and $h_{x2(y2)} = t_{x2(y2)}\sin(k_{x(y)})$.. The integration of 2D TPTI can be analytically solve based on residue theorem, which can be express as:

$$S_t = \begin{cases} \frac{(t_x-\Delta t_x)^2+(t_y-\Delta t_y)^2}{2}, & \Delta t_x > 0, \Delta t_y > 0 \\ \frac{(t_x+\Delta t_x)^2+(t_y+\Delta t_y)^2}{2}. & \Delta t_x < 0, \Delta t_y < 0 \end{cases} \tag{S8}$$

Therefore, the topological phase transition can be monitored by the peak of TPTI with the modulation of $\Delta t_{x(y)}$. The above discussion implies that the topological phase transition in 2D system can be directly observed by the photon propagation in real space.

In addition, we further discuss the relation between TPTI and the bandstructure. The real-space indicator TPTI characterizes the diffraction range of the photons in the waveguide lattice. For a fixed propagating distance, the value of TPTI is propotional to the group velocity of photons (the slope of the dispersion relation), which has been demonstrated in Eq.(S7). As demonstrated in Fig.2a in main text, topological phase transition is always accompanied with energy gap closing. At the band closing point, from Fig.2a in main text, we can see that the slope of the dispersion relation has a discontinuity, leading to the integral in Eq.(S7) (the value of TPTI) has a abrupt varaiation, which allows us to detect the topological phase transition.

In Eq.(S7), the method of TPTI assumes the propagating distance is longer enough, which limits the accuracy of the measured value of St. However, as discussed above, the topological phase transition point is manifested by the abrupt change of the value of TPTI, then topological phase transition indicator is quite robust to the accuracy of the value of TPTI (See Fig.3a in main text).

III. The properties of TPTI

As demonstrated in the main text, the system supports four topological phases with four wave polarization Q(see Fig.S2a). Based on the Eq.(2) in the main text, when $\Delta t_x < 0$ and $\Delta t_y < 0$, $Q = (0, 0)$, the topology in both direction are trivial. When $\Delta t_x > 0$ and $\Delta t_y > 0$, $Q = (\frac{1}{2}, \frac{1}{2})$, the topology in both direction are non-trivial. In addition, while for Δt_x or $\Delta t_y < 0$, the system is in the first-order topological insulator phase, which means only one direction in the system is non-trivial.

As demonstrated in section II, TPTI is related to the changes of topology for both x,y direction. In the process of transition from FOTI to SOTI or trivial phase (correspond to the vertical line or horizontal line in Fig.S2a,b), the wave polarization $Q = (\frac{1}{2}, 0)$ evolves to $Q = (\frac{1}{2}, \frac{1}{2})$ or $Q = (\frac{1}{2}, 0)$ evolves to $Q = (0, 0)$, which means that only one direction possess the phase transition and the phase in the other direction does not change. The TPTI peak in both cases is attributed to the single transition in only one direction. For comparison, in the process of transition from SOTI to trivial phase (correspond to the diagonal line in Fig.S2a,b), the wave polarization $Q = (\frac{1}{2}, \frac{1}{2})$ evolves to $Q = (0, 0)$, which means that both two direction simultaneously possess the phase transition with changes of both two wave polarization. Thus, the TPTI peak here can be regarded as the double topological transition for both two direction. As shown in Fig.S2b, TPTI peak associated with the transition from SOTI to trivial phase is higher than that associated with the transition from FOTI to SOTI or trivial phase.

Furthermore, the value of TPTI characterizes the diffraction range of the photons in the waveguide lattice. The higher TPTI means the photon in system has more free diffraction. We simulate the diffraction output photon patterns at three topological transition points(see inset of Fig.S2b), (i)$\Delta t_{x,y} = 0$, (ii)$\Delta t_x = 0, \Delta t_y = -0.2$, (iii)$\Delta t_x = 0.2, \Delta t_y = 0$ and a point at SOTI phase (iv)$\Delta t_x = 0.2, \Delta t_y = 0.2$. It is clear that, at the transition point from SOTI to trivial phase, the photons diffracts freely to the boundaries in both directions, which is far from the centre of the system. However, at the transition point from (ii)SOTI to FOTI or (iii)FOTI to trivial, the diffraction of photons is limited in y direction or x direction. At the point (iv)$\Delta t_x = 0.2, \Delta t_y = 0.2$, owing to the distortion and dimerization

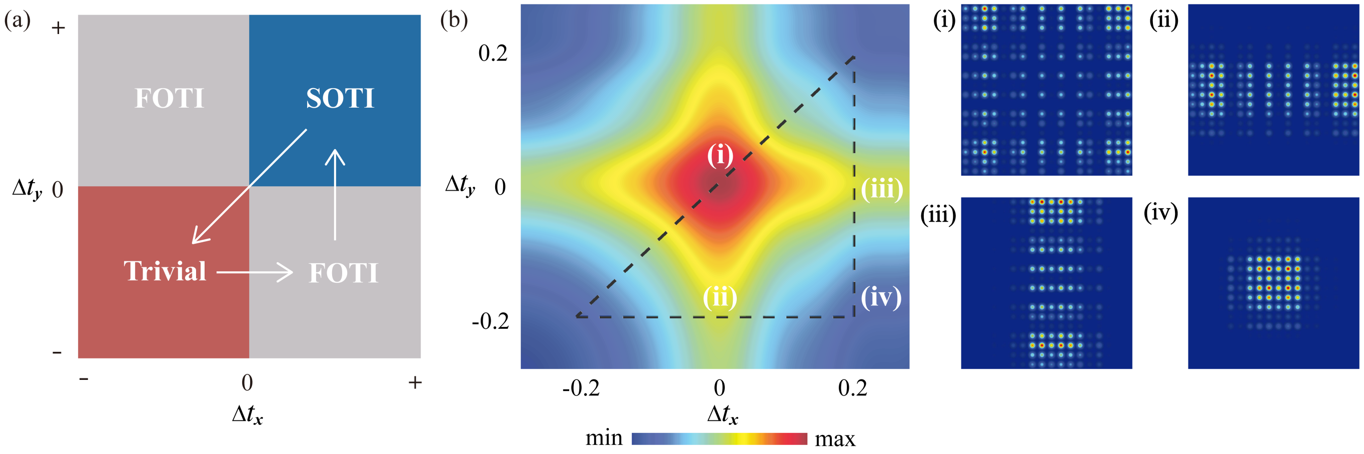

FIG. S2. **The properties of TPTI** (**a**)Topological phase diagram. The transitions are marked by white arrow. (**b**)The simulated results of TPTI. The simulated diffraction patterns are shown in inset (i)the transition point from SOTI to trivia phase, (ii)the transition point from FOTI to trivia phase, (iii)the transition point from FOTI to SOTI, (iv)the point with SOTI phase. Both simulations are in the same evolution length.

in both direction, the diffraction of photons is confined in the region surrounding the centre of the system.

IV. Creation and destruction of edge states along the phase transition

In the main text, we have demonstrated that we can observe the topological phase transition turning points via topological phase transition indicator with the excitation of bulk states. In this section, we focus on the edge states and discuss the creation and destruction of edge states during topological phase transition. Based on the Hamiltonian of our model shown in Eq(1) in the main text, we can get the energy structure for each distorted lattice and the combined local density of states(LDOS) of all edge modes.

As shown in Fig.S3(a), during the topological phase transition from FOTI to trivial phase via modulating Δt_x from 0.2 to -0.04 and keeping $\Delta t_y = -0.2$, the localized left and right edge modes appear and disappear, accompanied with the transition process. And the top and bottom boundaries remain unchanged with disappearing localized edge modes during the whole transition process. Therefore, the creation and destruction of left and right edge modes reflect the changes of topological phase transition from FOTI to trivial via modulating distortion along x direction and keeping y direction unchanging.

As shown in Fig.S3(b), during the topological phase transition from SOTI to FOTI phase via modulating Δt_y from 0.2 to -0.04 and keeping $\Delta t_x = 0.2$, the top and bottom edge keep localization in SOTI phase and then mix with the bulk when approaches to FOTI, accompanied with the transition process. And the left and right boundaries remain unchanged with localized edge modes during the whole transition process. Therefore, the creation and destruction of top and bottom edge modes reflect the changes of topological phase transition from SOTI to FOTI via modulating distortion along y direction and keeping x direction unchanging.

As shown in Fig.S3(c), during the topological phase transition from SOTI to trivial phase via simultaneously modulating both Δt_x and Δt_y from 0.2 to -0.04, the four edge modes (top, bottom, left and right) keep localization in SOTI phase and then gradually mix with the bulk when approaches to trivial phase. It shows that the simultaneous creation and destruction of top and bottom edge modes reflect the changes of topological phase transition from SOTI to trivial phase via modulating distortion both along x and y direction.

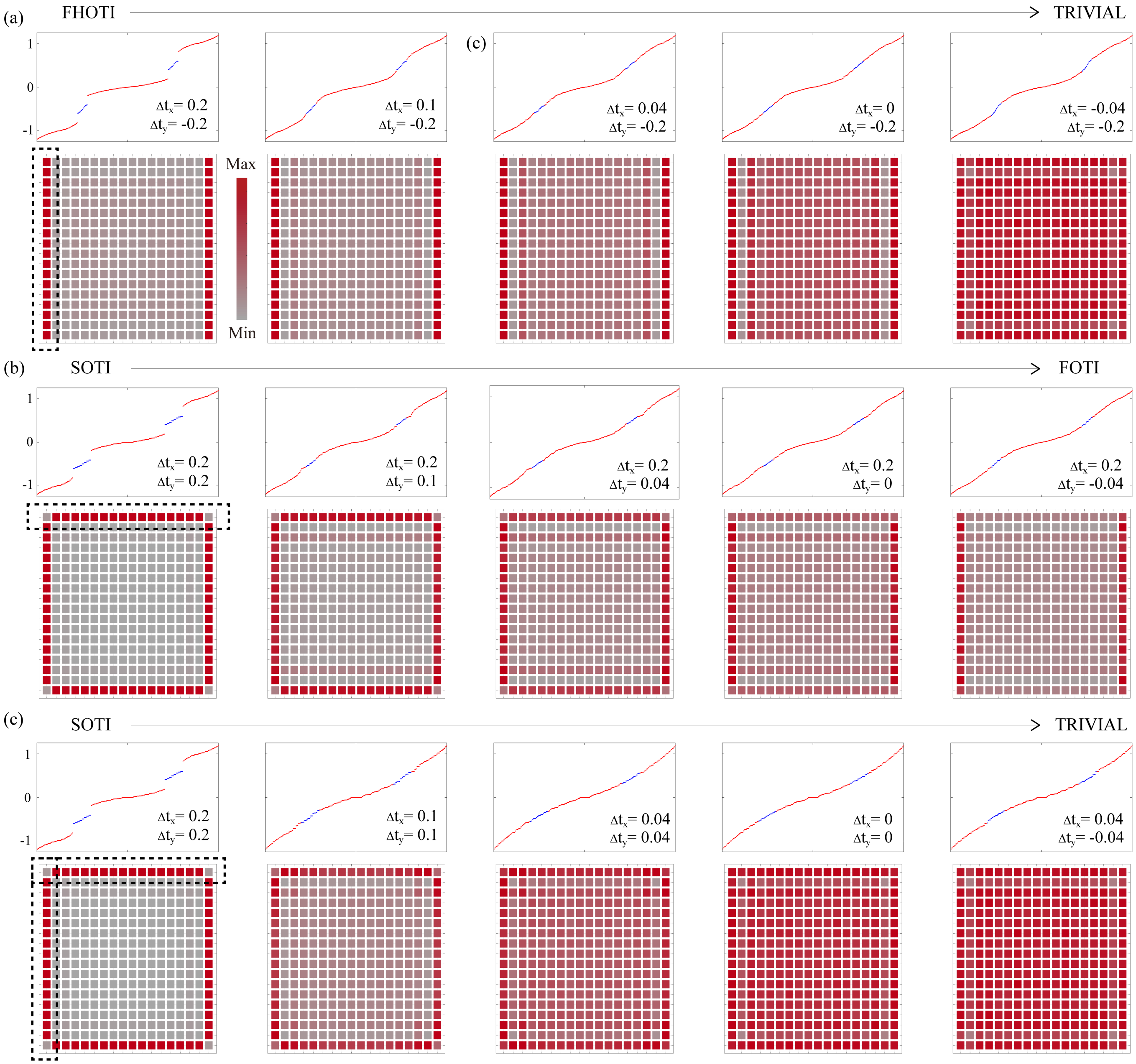

FIG. S3. **Energy structure and density distribution in distorted lattices** (**a**) Topological phase transition from FOTI to trivial phase. (**b**) Topological phase transition from SOTI to FOTI phase. (**c**) Topological phase transition from SOTI to trivial phase. The top panel shows the energy structure of the lattices. The blue dots indicate the edge modes. The bottom panel shows the combined LDOS of all edge modes. The black dash box shows the changed edge modes reflecting the topological phase transition, which we detect in experiment.
