# Supplementary figures and images for: Observing multifarious topological phase transitions with real-space indicator
Source: Nanophotonics. 2021 Nov 26;11(1):153–60. doi: 10.1515/nanoph-2021-0559 (PMC11501473; doi:10.1515/nanoph-2021-0559)

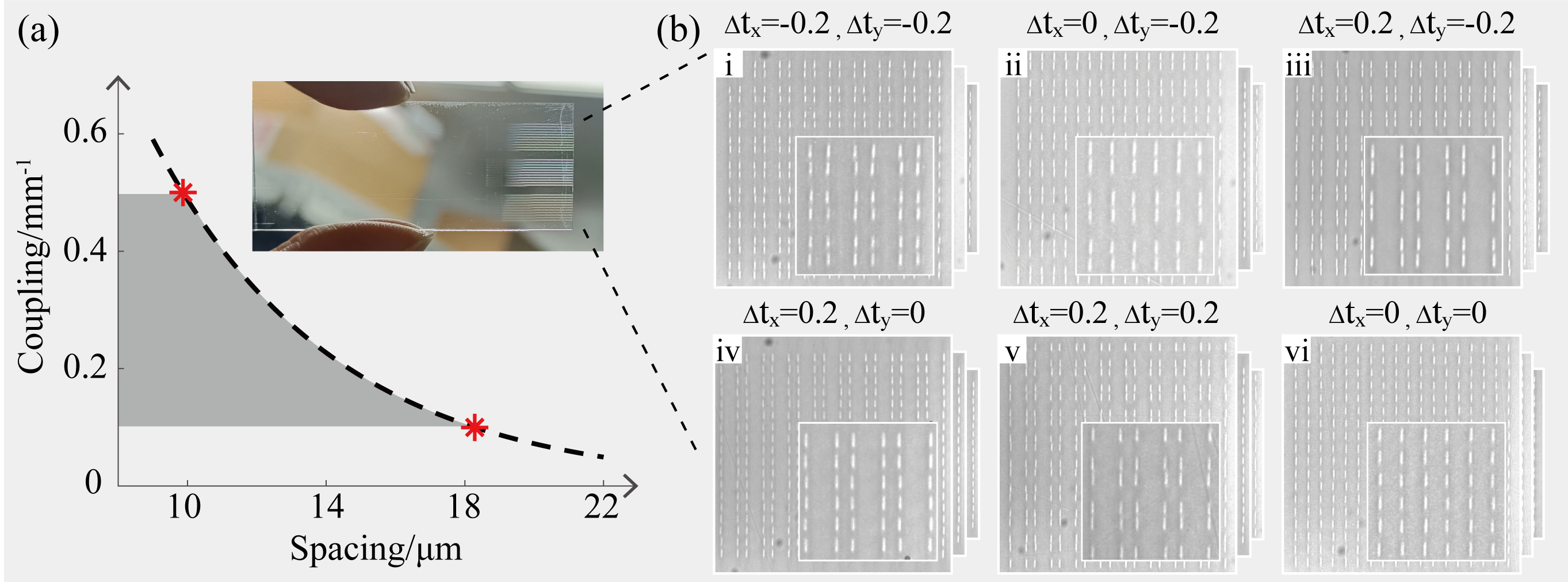

Supplement: Supplementary file 2 — Supplementary Material Details [file j_nanoph-2021-0559_suppl_002.zip › Supplementary Materials/figure_S1.png]

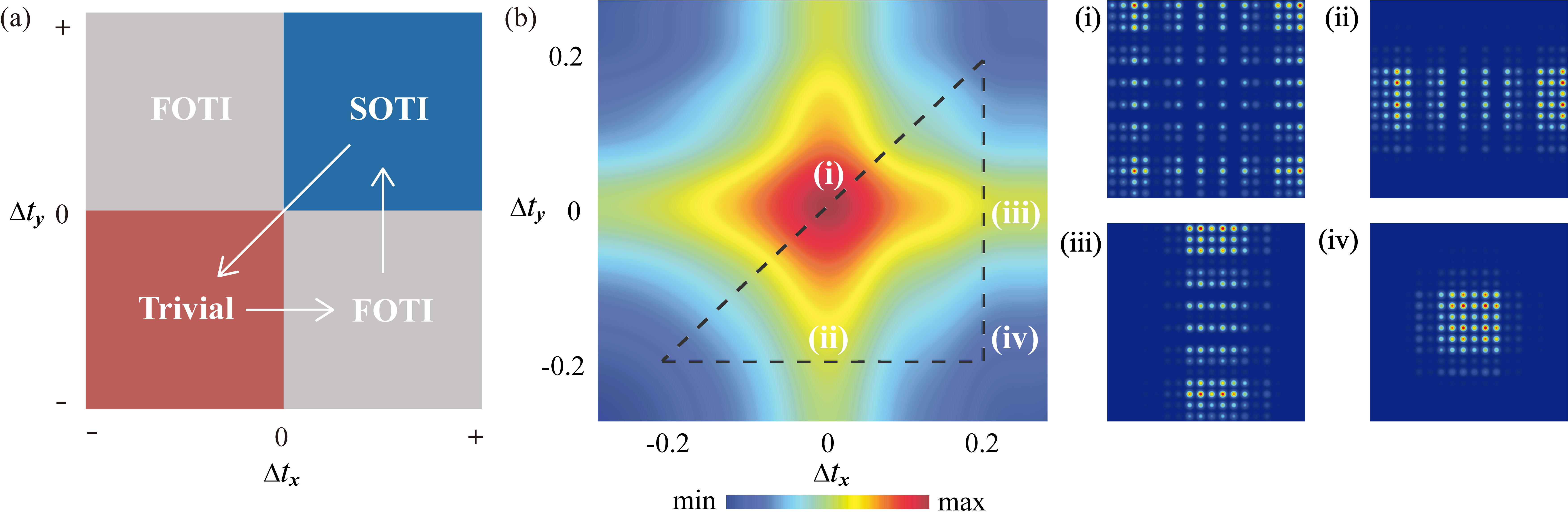

Supplement: Supplementary file 2 — Supplementary Material Details [file j_nanoph-2021-0559_suppl_002.zip › Supplementary Materials/figure_S2.png]

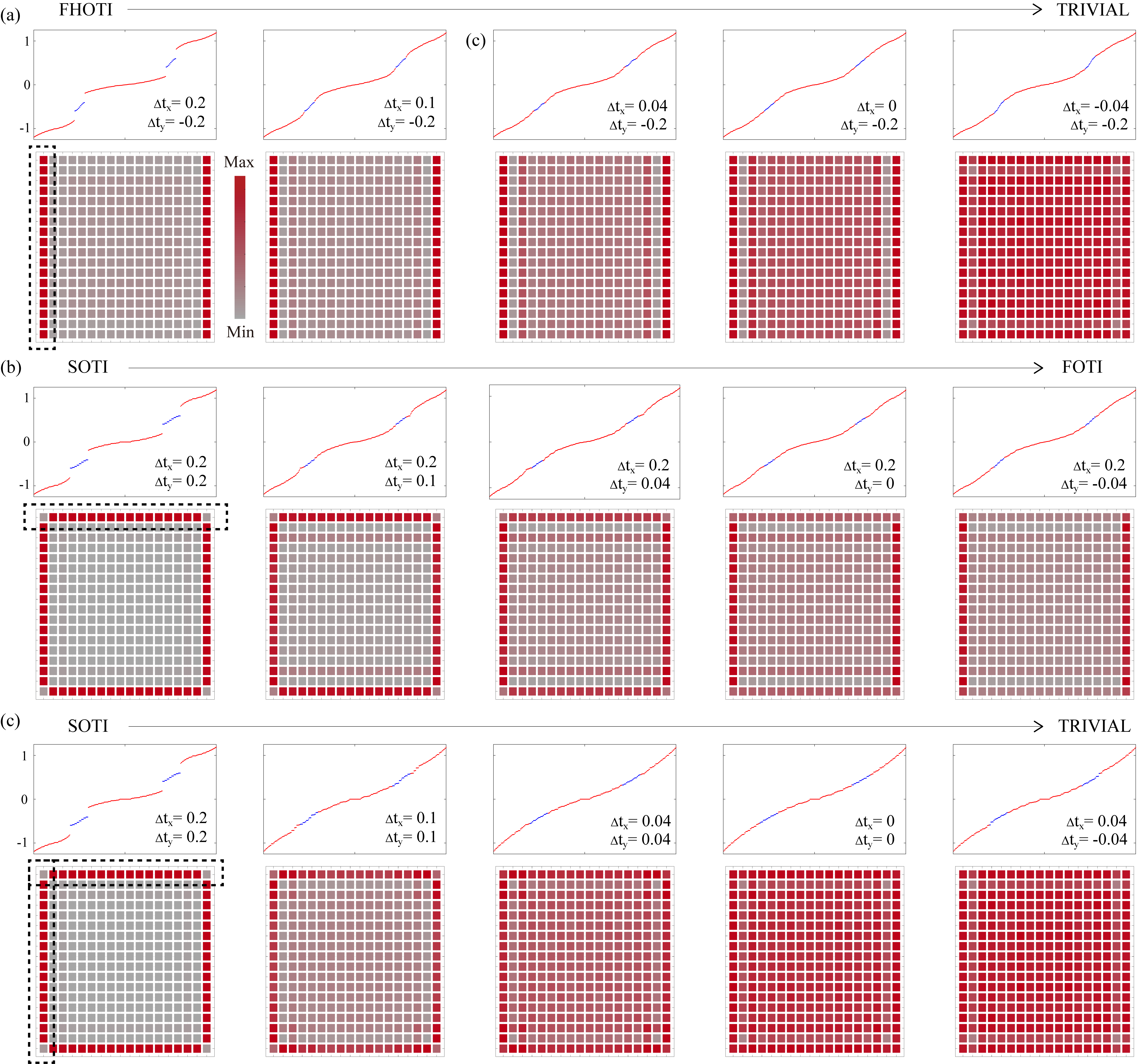

Supplement: Supplementary file 2 — Supplementary Material Details [file j_nanoph-2021-0559_suppl_002.zip › Supplementary Materials/figure_S3.png]
